# Identifying Defects with Guided Algorithms in Bragg Coherent Diffractive Imaging

**DOI:** 10.1038/s41598-017-09582-7

**Published:** 2017-08-30

**Authors:** A. Ulvestad, Y. Nashed, G. Beutier, M. Verdier, S. O. Hruszkewycz, M. Dupraz

**Affiliations:** 1 Materials Science Division, Argonne National Laboratory, Argonne, Illinois 60439 USA; 2 Mathematics and Computer Science, Argonne National Laboratory, Argonne, Illinois 60439 USA; 3 Univ. Grenoble Alpes, CNRS, Grenoble INP, SIMaP, F-38000 Grenoble, France; 4 Paul Scherrer Institute, Villigen, Switzerland

## Abstract

Crystallographic defects such as dislocations can significantly alter material properties and functionality. However, imaging these imperfections during operation remains challenging due to the short length scales involved and the reactive environments of interest. Bragg coherent diffractive imaging (BCDI) has emerged as a powerful tool capable of identifying dislocations, twin domains, and other defects in 3D detail with nanometer spatial resolution within nanocrystals and grains in reactive environments. However, BCDI relies on phase retrieval algorithms that can fail to accurately reconstruct the defect network. Here, we use numerical simulations to explore different guided phase retrieval algorithms for imaging defective crystals using BCDI. We explore different defect types, defect densities, Bragg peaks, and guided algorithm fitness metrics as a function of signal-to-noise ratio. Based on these results, we offer a general prescription for phasing of defective crystals with no *a priori* knowledge.

## Introduction

Understanding the defect influence on material properties is a topic of both scientific and technological importance^1^, relevant to strain-tolerant materials^2, 3^, ion intercalation^4, 5^, catalysis^6, 7^, and crystal growth^8, 9^. Consequently, much focus has been placed on understanding the functional links between the heterogeneous defect distribution and the resulting material properties. These research thrusts have been driven in part by the ability to image defects using electron microscopy^10^, x-ray scattering^11–14^, and, recently, coherent x-ray techniques^15–19^. Historically, electron microscopy has been used to observe dislocations due to its higher spatial resolution compared to x-ray techniques^20^. For example, the displacement field due to an individual edge dislocation was recently observed using electron microscopy techniques^10^. Extremely high resolution, transmission electron techniques typically require thin (<100 nm) samples and provide 2D images. Electron tomography can be used to visualize the 3D structure of samples^21, 22^, but is also limited to thin samples. The principle advantage of coherence-based x-ray techniques in the Bragg geometry is the ability to provide 3D images in reactive environments of defect networks for crystals of size 50–1000 nm^23, 24^. Bragg ptychography^16, 25^ translates the sample in the beam and uses probe position overlap to constrain the phase retrieval problem, and Bragg coherent diffractive imaging (BCDI) uses a finite support constraint (size estimate) for isolated nanocrystals and grains in polycrystalline films^26–28^. This work focuses on BCDI. While there has been success imaging defects in a variety of systems and environments^17, 18, 26, 27^, there has yet to be a systematic investigation of optimal phase retrieval procedures for recovering defect networks.

Phase retrieval in coherent diffraction imaging (CDI) is a non-convex optimization problem in which convergence to the true solution (image) is not guaranteed. Many algorithms have been proposed^29^ and the two most commonly used are the error reduction (ER) algorithm^30^ and the Hybrid Input-Output (HIO) algorithm^31^. The most common practice for BCDI phase retrieval is to alternate between ER and HIO while utilizing the Shrinkwrap algorithm^32^ to update the support. This procedure was improved upon recently by the utilization of guided algorithms^33, 34^ in which phase retrieval is performed a number of times and the results are combined before being used as the initial start for another set of iterations. Note that some past approaches have focused on modifying the algorithms themselves^35, 36^ while in guided algorithms the algorithm starting point is influenced but the algorithms are unmodified. Guided phase retrieval algorithms have produced consistent experimental results for dislocation imaging^17, 19, 26, 27^. However, because there are no convergence guarantees^37^, it is unclear how and under what conditions images of dislocations are accurately recovered. In particular, the best method for generating a suitable starting point after each generation of the guided algorithm under different signal-to-noise conditions remains unexplored and is the focus of this work. Past studies have focused on noise effects in 2D transmission ptychography^38–40^, which is not sensitive to crystallographic defects.

**Figure 1:**
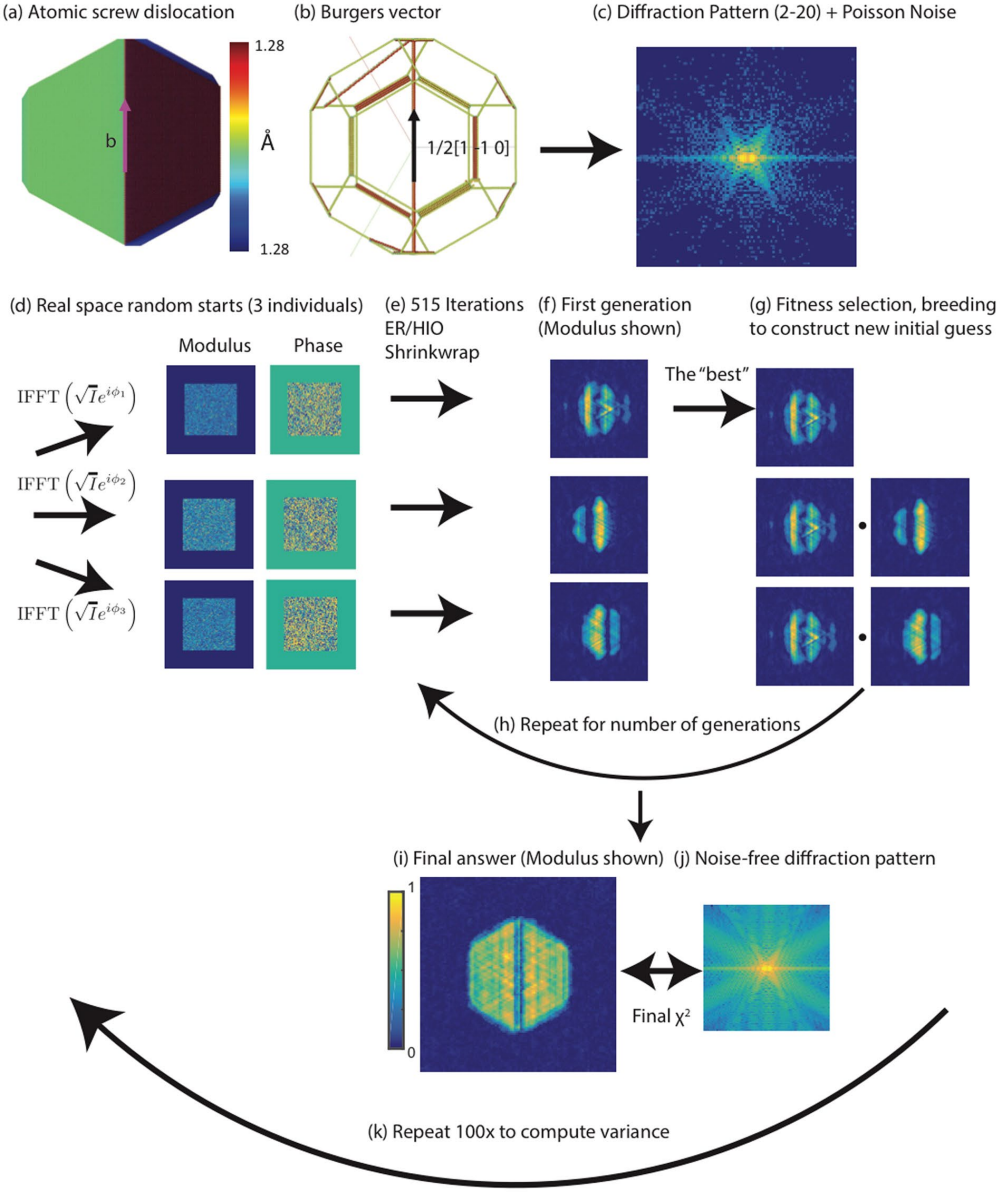
Flow diagram for the guided phase retrieval algorithm. **(a)** 2D view of the crystal with a perfect screw dislocation. **(b)** 3D view of the dislocation showing the Burgers vector (1/2 [1–1 0]). **(c)** The (2–20) diffraction pattern computed from the atomic arrangement in **(a,b)** with Poisson noise added to simulate experimental data. **(d)** The noisy data is used to generate three individual real space random starts by using different random phases and the inverse Fourier transform. **(e)** 515 total iterations alternating between ER and HIO with Shrinkwrap are performed for each individual to recover the real space image shown in **(f)**. **(g)** The best image from the first generation of individuals is selected according to a fitness metric. It is then bred into the other individuals. The total number of individuals is always fixed at three. **(h)** The bred individuals are fed back into **(e–g)** for a number of generations. The final answer (modulus shown) **(i)** is compared against the noise-free diffraction data **(j)** to compute the final modulus error. **(k)** The sequence **(c–j)** is repeated 100x to estimate the variance and success rate for a given fitness metric. We keep all algorithm parameters fixed except for the metric used to select the fittest individual from a generation. 3D reconstructions were performed although 2D cross-sections are shown.

In order to offer guidelines for successful phase retrieval of defective crystals, we have undertaken numerical simulation under different conditionsusing GPU-accelerated code in Matlab^54^﻿. Bragg coherent diffraction data from defective crystals were simulated under the kinematic approximation using an atomistic model assuming a plane wave illumination^41^. The crystals considered are approximately 30 × 30 × 30 nm^3^ in real space and contain approximately 10^6^ atoms, with a real space pixel size of 0.7 nm. Note that this pixel size is smaller than those experimentally obtained, which are typically 4 nm − 14 nm. This does not impact the results as the crystal size can be scaled appropriately without loss of generality. Finally, it has been shown that the oversampling ratio plays a role in phase retrieval convergence^42^. Typical experiments are done with an oversampling ratio of 3 and consequently we kept the oversampling fixed at 3 throughout the simulations. We first discuss the guided algorithm as outlined by the flow diagram in Fig. 1.

We consider various types of dislocations and configurations in nanocrystals, one of which is shown in Fig. 1a. The dislocation is oriented such that the Burgers vector is ½[1–1 0] (Fig. 1b). Coherent diffraction data at the (2–20) peak is calculated under the kinematic approximation by summing the amplitudes from each atom with its phase factor^41^. Note that x-ray diffraction at a particular Bragg peak is sensitive to a dislocation if the displacement field generated by the dislocation has a non-zero projection onto the particular Bragg peak. By measuring three independent Bragg peaks, the complete vector displacement field can be imaged and all of the dislocation orientations are exactly determined^43^. The 3D data set is 128 × 128 × 128, an oversampling ratio of 3, and a reciprocal space step size (pixel size) of 0.011 nm^−1^ ^41^. Poisson noise is then applied to each pixel after the data is rescaled assuming a maximum number of photons in the central pixel (subsequently referred to as “photon max”) (Fig. 1c). This noisy data is used to generate three individuals in the first generation by inverse Fourier transforming the square root of the noisy data with different sets of random phases (Fig. 1d). This ensures different starting points for the algorithm. The algorithm is then run: 515 total iterations with 40 iterations of ER followed by 40 iterations of HIO with Shrinkwrap^32^ every 5 iterations (Fig. 1e). The algorithm terminates before finishing the final 40 ER iterations. The HIO feedback parameter is 0.9. The Shrinkwrap is done every 5 iterations during both algorithms using a Gaussian function with a standard deviation of 1 pixel and a threshold of 0.1. Different fitness metrics are then computed from the images (Fig. 1f). Different fitness metrics are then used to evaluate the images (Fig. 1f), listed in Table 1:

**Table 1:** Fitness metrics used during the test of the guided algorithms.

Name	Equation	Captures
Chi	$\sum _{{\boldsymbol{q}}}\frac{{(|{\rm{FFT}}(\rho ({\boldsymbol{r}}))|-\sqrt{I({\boldsymbol{q}})})}^{2}}{I({\boldsymbol{q}})}$	Agreement to measured (noisy) data. The minimum in this metric is selected.
Sharp	$\sum _{{\bf{r}}}{|{\rm{\rho }}({\bf{r}})|}^{4}$	Uniformity of reconstructed amplitudes. The minimum in this metric is selected.
Sharp norm	${(\sum _{{\boldsymbol{r}}}{|\rho ({\boldsymbol{r}})|}^{1/4})}^{4}$	Based on the Sharp metric but preserving the units of $\rho$. The minimum in this metric is selected.
Max Volume	$\sum _{{\boldsymbol{r}}}|\rho ({\boldsymbol{r}})|/\,{\rm{\max }}|\rho ({\boldsymbol{r}})|$ with $|\rho ({\boldsymbol{r}})| < 0.2=0$, $|{\rho }({r})|\ge {\rm{0}}\mathrm{.2}=1$	Total volume of the reconstructed crystal. The maximum in this metric is selected.

The fitness metrics other than Chi in Table 1 are designed to promote desirable features in the final image, namely the uniformity of amplitudes (Sharp and Sharp norm) and the size of the crystal (Max Volume). Please see Supplementary Fig. 1 for a demonstration of how selecting the minimum Sharp metric value promotes amplitude uniformity. It has been observed that the ER/HIO/Shrinkwrap combination can aggressively shrink the crystal volume and become trapped in a local minimum that prevents convergence to the true solution^17^. This is because Shrinkwrap is effectively a sparsity promoting operation^44^. The Max Volume metric is designed to favor “anti-sparsity” directly by choosing the largest crystal, which is defined as the crystal with the most nonzero pixels after thresholding the density at 20% of its maximum. The Sharp and Sharp norm promote anti-sparsity indirectly through amplitude uniformity. Finally, we note that the fitness metrics are only used to influence the initial starting point of the reconstruction algorithms and not the algorithms themselves, as in previous approaches^35, 36^.

The fittest individual is selected according to one of the metrics in Table 1 and then bred into the other individuals via ${\rho }_{new,i}=\sqrt{{\rho }_{i}{\rho }_{best}}$ after each individual is centered in the computational array, has phase ramps removed, and has the same global phase offset applied (Fig. 1g). The number of individuals is always fixed at 3. In this work, we did not explore different breeding methods. After breeding, the entire procedure is repeated for a number of generations (3 in this work) (Fig. 1h). The final image (Fig. 1i) is then selected after 3 generations of 3 individuals. The final modulus error is computed with respect to the noise-free diffraction pattern as opposed to the noisy data as this is a better measure of agreement (Fig. 1j). The entire procedure is repeated 100x (Fig. 1k) to assess the success rate of each fitness metric. We discuss the results of this procedure for the case of a perfect edge dislocation in a facetted nanocrystal with Wulff geometry. From this atomic model, coherent Bragg diffraction about a (1–11) Bragg peak was calculated and used to test the guided algorithm fitness metrics.

**Figure 2:**
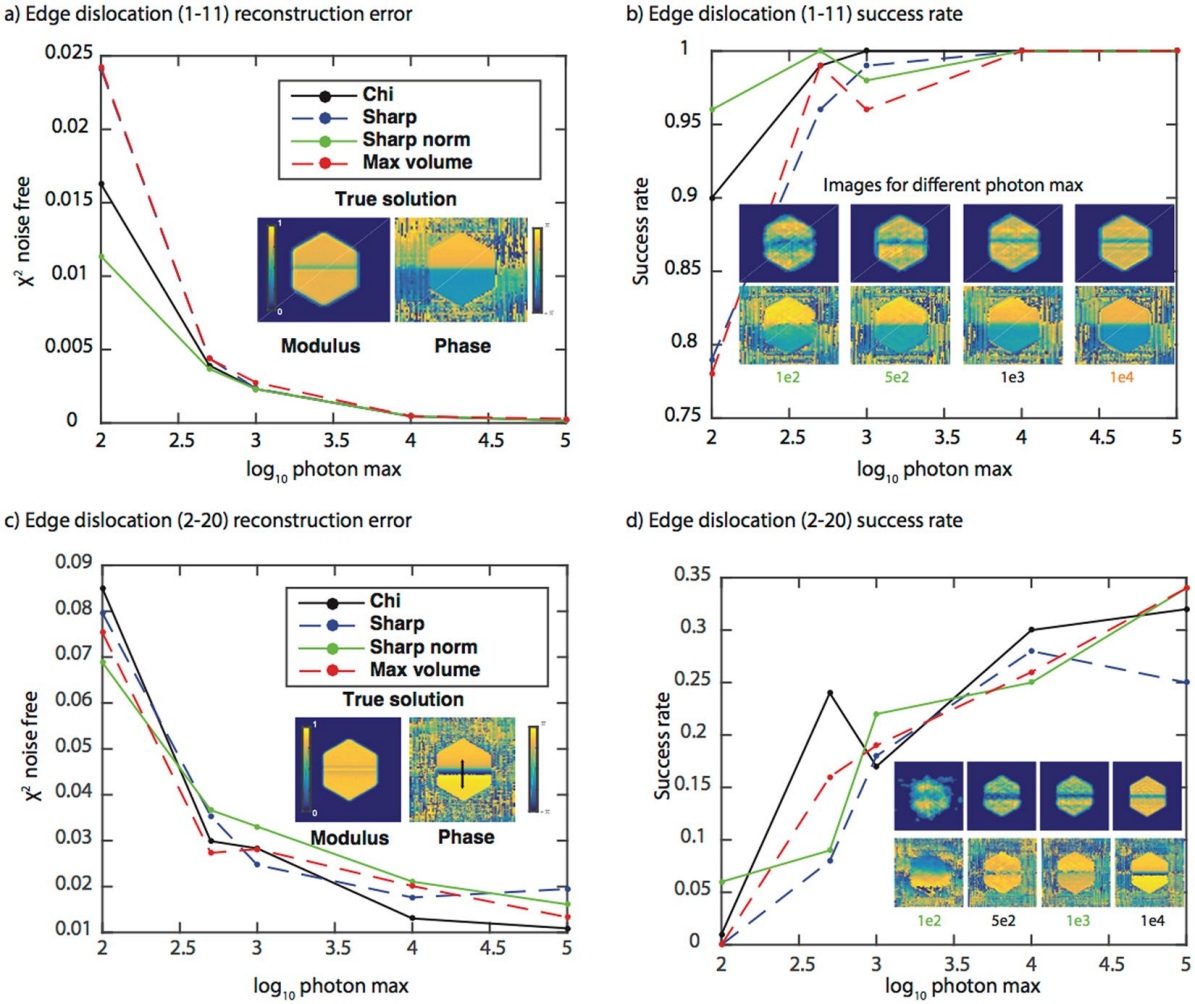
Fitness metric results for a perfect edge dislocation measured at a (1–11) and a (2–20) Bragg peak. The (1–11) Bragg peak: **(a)** A plot of the average modulus error vs. log_10_ of the photon max for the four fitness metrics. The modulus error (χ^2^) is computed with respect to the noise-free data and averaged over the 100 trials. A 2D cross-section of the true solution modulus and phase is shown as an inset. The colorbars apply to all modulus and phase cross-sections in the figure. **(b)** A plot of the success rate (over 100 trials) for the four fitness metrics as a function of photon max. The success rate is defined as the number of successful trials, which is defined through a correlation to the true solution, divided by the total number of trials. The inset shows the best-correlated reconstructed image obtained using the metric with the highest success rate. The color of the photon max number at the bottom of the image corresponds to the most successful metric. The metrics with the highest success rate percentages are: 1e2 – Sharp norm, 5e2 – Sharp norm, 1e3 – Chi, 1e4 – all are equivalent (hence the orange color), 1e5 – all equivalent (not shown). Note that 3D reconstructions were performed although 2D cross-sections are shown. **(c)**–**(d)** the same as **(a)**–**(b)** but for the (2–20) Bragg peak.

Figure 2 shows the results for the four different fitness metrics in reconstructing a perfect edge dislocation measured at a (1–11) and a (2–20) Bragg peak with various levels of noise. The Sharp norm metric performs best (has the lowest modulus error averaged over 100 trials) at low signal-to-noise ratios (100 and 500 photon max) while the metrics are essentially indistinguishable at 1e3, 1e4, and 1e5 photon max (Fig. 2a). The fact that a metric other than Chi performs best (has a better agreement to the true data) at low photon max likely stems from the fact that the Chi metric will favor individuals that best match the noisy data. Maximum-likelihood refinements have been introduced in transmission ptychography to improve resolution in the presence of noise^45, 46^. However, we found that the maximum-likelihood metric performed worse than the four metrics discussed in Fig. 2 for this particular case and consequently we do not consider it further. At high signal to noise ratios, the effect of noise is minimal and all metrics perform equally well.

The modulus metric is useful as one measure of the reconstructed image accuracy but more important is whether or not the image accurately reflects the interesting structural features (dislocation number, arrangement, etc.). To this end, we evaluated the real-space modulus correlation coefficient^47^ of all 100 3D images to determined a success rate. A successful reconstruction was deemed to be one with a correlation coefficient to the true solution of greater than 85% (Supplementary Fig. 2). We found the distribution of correlation coefficients to be bimodal, consisting of either successful image reconstruction or very poor ones. Figure 2b shows the success rate for all four fitness metrics as a function of photon max, and the most highly correlated image from the most successful metric that was reconstructed at each photon max. Even with a maximum of only 100 photons in the diffraction pattern, the crystal image bears a strong similarity to the true image. This is reassuring given that experimental data sets often contain >10^5^ max photons. Increasing photon max tends to improve the amplitude uniformity while leaving the phase relatively unchanged. This suggests that amplitude inhomogeneity in low signal-to-noise measurements should be interpreted cautiously while the phase information is more robust. Finally, we note that with 1e4 photon max all metrics have a 100% success rate. To assess the robustness of these conclusions, we investigated the same four fitness metrics for the case of the same edge dislocation measured at a (2–20) Bragg peak (Fig. 2c).

Figure 2c shows the results for the four different fitness metrics in reconstructing the same edge dislocation measured at a (2–20) Bragg peak with various levels of noise. The Sharp norm metric performs best (has the lowest modulus error) at 100 photon max while Chi performs best at higher photon max. One large difference between Fig. 2a and c is the agreement to the true data. The edge dislocation reconstructed at the (2–20) peak shows ~100x higher modulus error at 1e5 photon max compared to the (1–11) measurement. The success rate for all signal to noise ratios is also much worse (Fig. 2d) compared to Fig. 2b. In all cases at the (2–20) peak, no metric succeeds more than 35% of the time. This fact, combined with the previous discussion of the quantitative errors, leads us to conclude that the success of phase retrieval depends on the phase distribution of a given defect at different Bragg conditions. The phase distribution expected in the image at a given Bragg peak is equal to the dot product of the atomic displacement field and the scattering vector of the measured Bragg peak:

$$\varphi ={\boldsymbol{u}}({\boldsymbol{r}})\cdot {{\boldsymbol{G}}}_{hkl}$$

with $\varphi$ the reconstructed phase, ${\boldsymbol{u}}({\boldsymbol{r}})$ the atomic displacement field, and ${{\boldsymbol{G}}}_{hkl}$ the scattering vector corresponding to the (hkl) Bragg peak. For the (1–11) peak from a face centered cubic crystal ${{\boldsymbol{G}}}_{111}=\frac{2\pi }{{d}_{1-11}}$ with ${d}_{111}=$ $\frac{a}{\sqrt{{1}^{2}+{(-1)}^{2}+{1}^{2}}}=a/\surd 3$and therefore a phase value of 2π corresponds to a physical displacement of $a/\surd 3$. At a higher order Bragg peak such as (2–20), ${{\boldsymbol{G}}}_{2-20}=\frac{2\pi }{{d}_{2-20}}$ with ${d}_{2-20}=\frac{a}{\sqrt{{2}^{2}+{(-2)}^{2}+{0}^{2}}}\,=a/\surd 8$ and therefore a phase value of 2π corresponds to a physical displacement of $a/\surd 8$. Therefore, when the physical displacement induced by the dislocation (${\boldsymbol{u}}({\boldsymbol{r}})$) projected onto the Bragg peak is the same, more phase wraps (regions of phase from 0 to 2π) are required for higher order peaks (in this case the (2–20) peak). The inset in Fig. 2c shows the phase ramp present in the (2–20) image that is not present in the (1–11) image. Reconstruction algorithms have trouble dealing with multiple phase wraps^48–50^. To explore the influence of the dislocation type, we performed the same simulations on a screw dislocation measured about a (2–20) peak.

**Figure 3:**
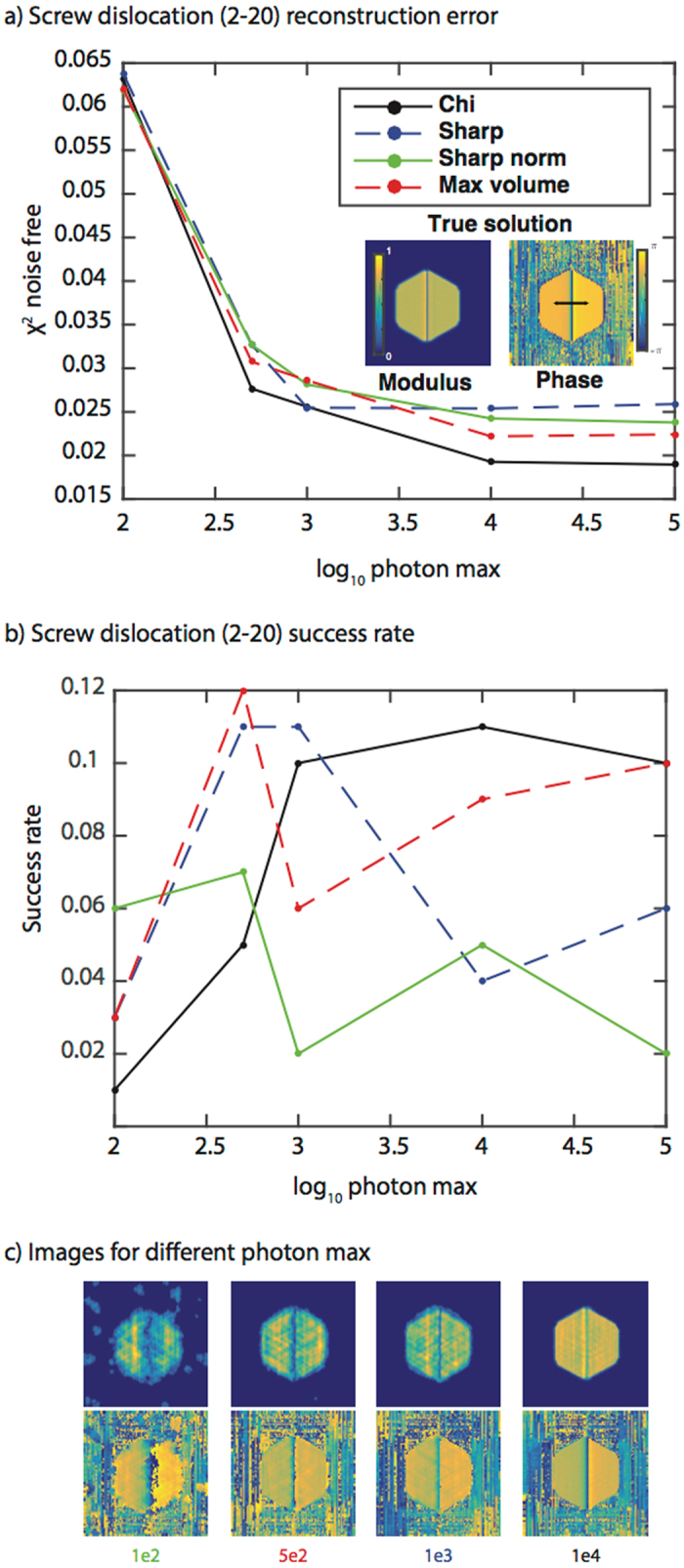
Fitness metric results for a perfect screw dislocation measured at a (2–20) Bragg peak. **(a)** A plot of the modulus error vs. log_10_ of the photon max for the four fitness metrics. The modulus error (χ^2^) is computed with respect to the noise-free data and averaged over the 100 trials. A 2D cross-section of the true solution modulus and phase is shown as an inset. The colorbars apply to all modulus and phase cross-sections in the figure. The black arrow shows the phase wrap direction in the image. **(b)** A plot of the success rate (over 100 trials) for the four fitness metrics as a function of photon max. The legend is the same as in **(a)**. **(c)** The best-correlated images obtained using the metric with the highest success rate for different photon max. The color of the photon max number at the bottom of the image corresponds to the most successful metric. The metrics with the highest success rates are: 1e2 – Sharp norm, 5e2 – Max volume, 1e3 – Sharp, 1e4 – Chi, 1e5 – Chi/Max volume (not shown). Note that 3D reconstructions were performed although 2D cross-sections are shown.

Figure 3 shows the results for the four different fitness metrics in reconstructing a perfect screw dislocation measured at a (2–20) Bragg peak with various levels of noise. The Max volume metric performs best (has the lowest modulus error) at 100 photon max while Chi performs best at higher photon max (Fig. 3a). The success rate is low (Fig. 3b), but the reconstructions that are successful are still similar to the true image (Fig. 3c). In these cases, the most successful metrics are: 1e2 – Sharp norm, 5e2 – Max volume, 1e3 – Sharp, 1e4 – Chi, 1e5 – Chi (not shown). Consistent with Fig. 2b, increasing the photon max primarily improves the amplitude. We considered the same screw dislocation measured at a (1–11) peak (Supplementary Fig. 3). The differences between the (1–11) Bragg peak and the (2–20) Bragg peak are similar to the differences for the edge dislocation discussed previously (Fig. 2).

We also investigated other dislocation types. We considered a relaxed screw dislocation measured at a (1–11) peak (Supplementary Fig. 4), a relaxed screw dislocation measured at a (2–20) peak (Supplementary Fig. 5), a relaxed edge dislocation measured at a (1–11) peak (Supplementary Fig. 6), a relaxed edge dislocation measured at a (2–20) peak (Supplementary Fig. 7), a Frank loop dislocation array measured at a (11–1) peak (Supplementary Fig. 8), and a Frank loop dislocation array measured at a (2–20) peak (Supplementary Fig. 9 **)**. In these supplementary figures, the error bars correspond to two standard deviations of the modulus error over the 100 trials. As before, both the modulus metric and success rate are shown for the four metrics as a function of max photon number. These results support the general tendencies: Sharp norm performs best at 100 photon max, the metrics are indistinguishable at high photon max, and higher index Bragg peaks result in poorer quality images than their lower index counterparts. Additionally, we find that the relaxed dislocations (dissociated into Shockley partials^41^) tend to be easier to reconstruct as they have less abrupt boundaries of displacement than their unrelaxed counterparts. Thus, the relaxed dislocations are easier to reconstruct for the same reason as discussed above for comparing high and low index Bragg peaks.

We have so far discussed only single dislocations. Real crystals often contain many defects arranged in complex networks. To investigate this case, we performed the same phase retrieval procedure discussed previously on diffraction data from simulations of a Ni film that nucleated multiple dislocations in response to nanoindenting^41, 51^. BCDI was recently developed for individual grain imaging in polycrystalline films^28, 52^ and thus the situation considered here is that the Ni film grain is smaller than the beam size.

**Figure 4:**
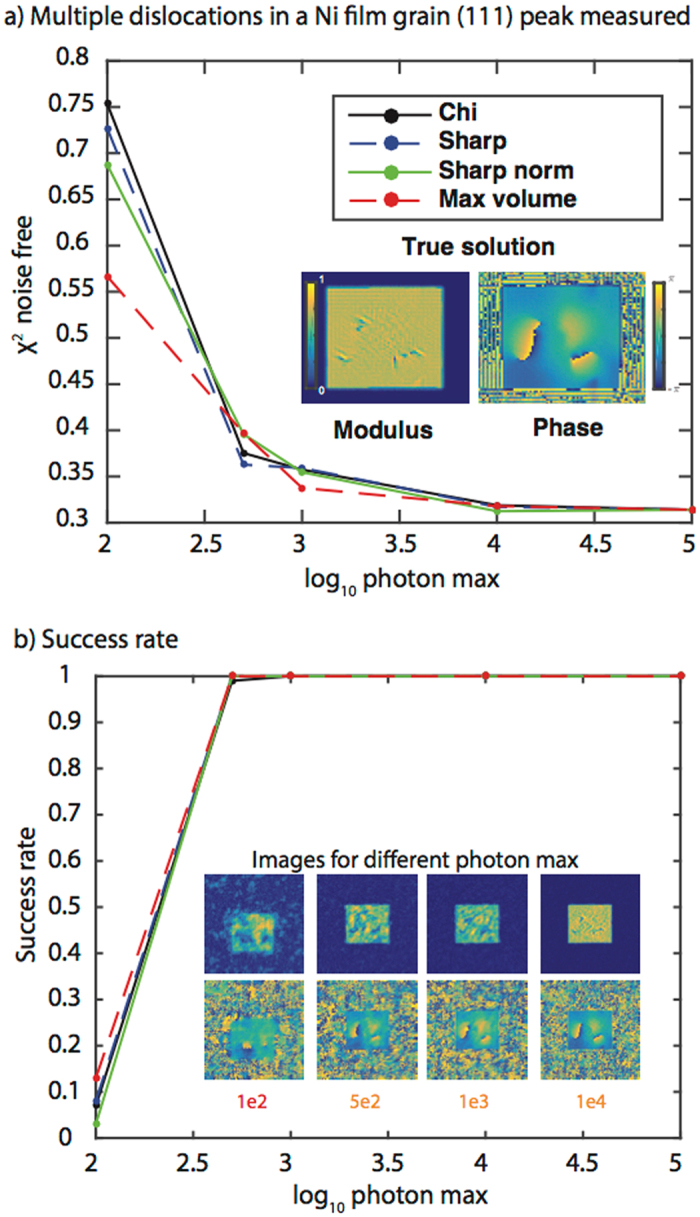
Fitness metric results for a dislocation network in a Ni film measured at a (111) Bragg peak. **(a)** A plot of the modulus error vs. log_10_ of the photon max for the four fitness metrics. The modulus error (χ^2^) is computed with respect to the noise-free data. A 2D cross-section of the true solution modulus and phase is shown as an inset. The colorbars apply to all modulus and phase cross-sections in the figure. **(b)** A plot of the success rate (over 100 trials) for the four fitness metrics as a function of photon max. The inset shows the best-correlated reconstructed image obtained using the metric with the highest success rate. The color of the photon max number at the bottom of the image corresponds to the metric. The metrics with the highest success rates are: 1e2 – Max volume, 5e2 – Sharp, 1e3 – Sharp, 1e4 – all are equivalent (hence the orange color), 1e5 – all equivalent (not shown). Note that 3D reconstructions were performed although 2D cross-sections are shown.

Consistent with Figs 2 and 3, a metric other than Chi performs best at low photon max while at high photon max all metrics perform similarly (Fig. 4a). For 100 photon max, the Max volume metric has both the best modulus error and the highest success rate (Fig. 4b). Two sets of dislocations can be identified albeit the extent of the dislocation lines and their location in the film are not accurately reconstructed. At 500 photon max, the images are improved and all metrics recover the correct dislocation distribution with the Sharp metric succeeding most frequently (Fig. 4b). At higher photon max the fitness metrics become indistinguishable and all accurately recover the dislocation network. Consistent with previous results (Figs 2 and 3 **)**, increasing photon max mostly affects the amplitude homogeneity. While it has been argued that BCDI cannot reconstruct many dislocations, these simulations show that, provided the dislocation spatial separation is not below the image resolution, BCDI can recover the dislocation network provided the max photon number is equal to or greater than 500. To show the robustness of these conclusions, we performed the same simulations on two other Brag reflections from the same dislocation distribution (Supplementary Figs 10–11) and find similar results.

The important conclusions of this work are as follows. First, the use of fitness metrics that promote “anti-sparsity” for low signal-to-noise measurements will outperform (have lower modulus errors relative to the true data and have a higher success rate) the traditional Chi metric in reconstructing crystals with both single and multiple defects. Second, all metrics perform similarly for photon max greater than or equal to 1000, which leads us to have a high confidence in the experimental reconstructions of dislocation networks thus far reported. Third, images will tend to be more accurate and easier to reconstruct when lower order Bragg peaks are measured, though sensitivity to small displacements is sacrificed. Finally, we find that BCDI phase retrieval algorithms can handle dislocation networks provided that dislocation cores are separated spatially. In light of these conclusions, we offer the following general prescription for phase retrieval of a suspected defective crystal using a guided algorithm approach: Choose a low order Bragg peak, and if the max photons in the image are low, the Chi metric should be avoided in favor of the Sharp norm or Maximum volume metric. It is important to point out the limitations of the present study as well. We have considered four particular metrics, 13 simulations at different Bragg peaks from a variety of dislocation distributions, a fixed breeding step, and a fixed oversampling ratio. However, we have used parameters close to those of a typical experiment. Thus, these general results will be useful in reconstructing defective crystals in a variety of interesting applications, especially when the specimen of interest is weakly scattering^53^. Finally, the results of these investigations may find uses in phase retrieval in other applications, including x-ray ptychography^54^.

### Data Availability

The data reported in this paper are available upon request. All code, including the reconstruction algorithm, is also available upon request.

## Electronic supplementary material

Supplementary Information
