# Supplementary material for: Identifying Defects with Guided Algorithms in Bragg Coherent Diffractive Imaging
Source: Sci Rep. 2017 Aug 30;7:9920. doi: 10.1038/s41598-017-09582-7 (PMC5577107; doi:10.1038/s41598-017-09582-7)
Supplement: Supplementary file 1 — Supplementary Information [file 41598_2017_9582_MOESM1_ESM.pdf]

Identifying Defects with Genetic Algorithms in Bragg Coherent Diffractive Imaging

A. Ulvestad[1]*, Y. Nashed[2], G. Beutier[3], M. Verdier[3], S. O. Hruszkewycz[1], and M. Dupraz[4]

[1]Materials Science Division, Argonne National Laboratory, Argonne, Illinois 60439, USA

[2]Mathematics and Computer Science, Argonne National Laboratory, Argonne, Illinois 60439, USA

[3]Univ. Grenoble Alpes, CNRS, Grenoble INP, SIMaP, F-38000 Grenoble, France

[4]Paul Scherrer Institute

*aulvestad@anl.gov

Supplementary Materials

Fig S1-S11

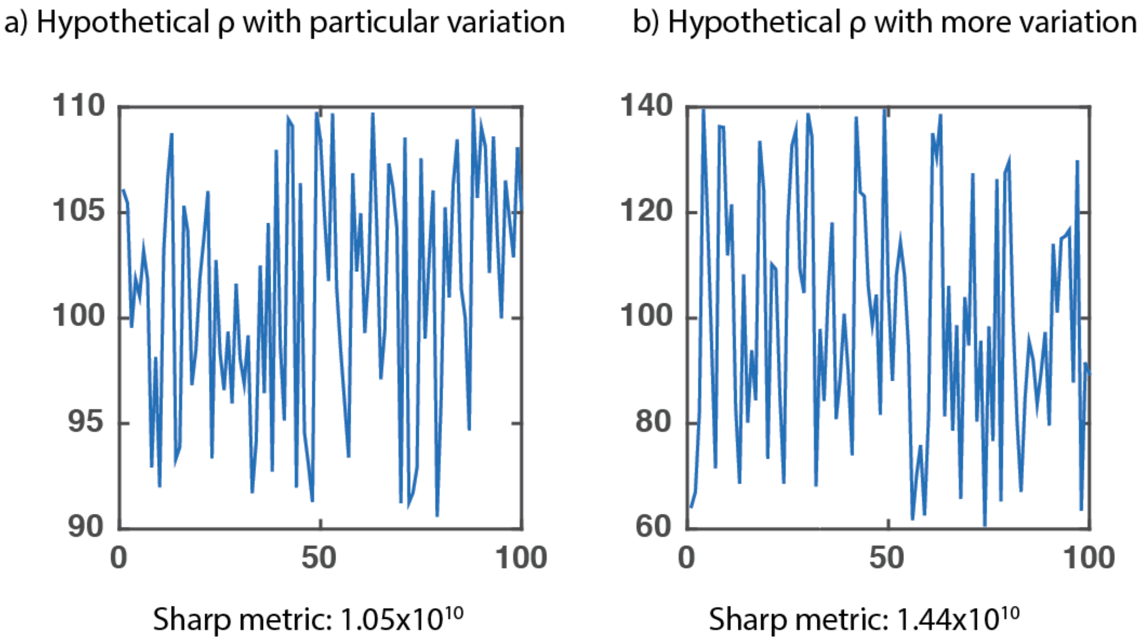

Figure S1. Example values for the modulus of the reconstructed image generated using random numbers centered on 100 with different interval widths. (a) shows the modulus values that vary less than in (b). The Sharp metric values for each are shown below the figure. Choosing the minimum in the Sharp metric corresponds to choosing the smoother amplitude distribution.

a) Screw dislocation, (1-11) peak, modulus cross-sections for selected trials

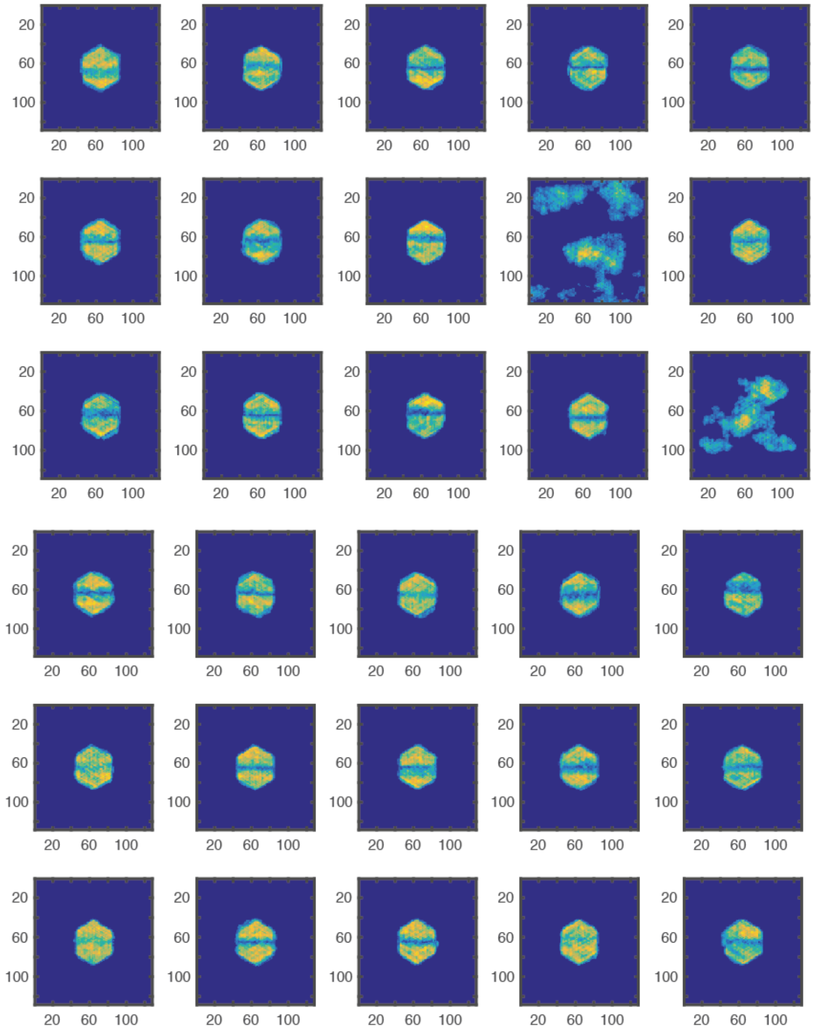

b) The corresponding Chi metric for all 100 trials

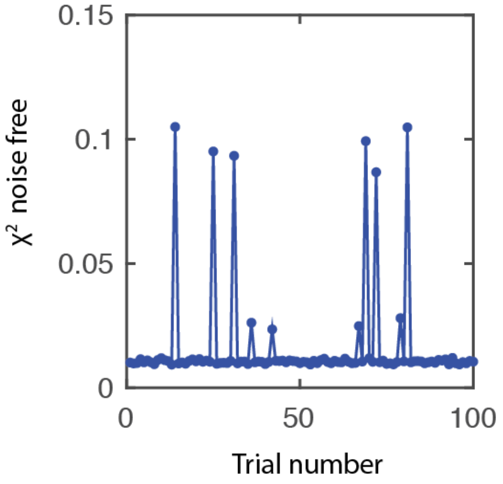

c) The corresponding Modulus correlation

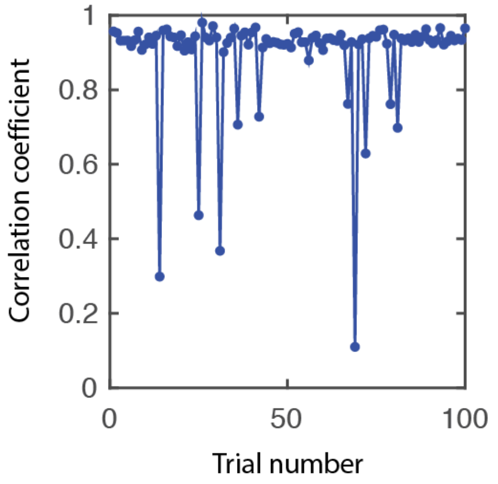

Figure S2. A perfect screw dislocation measured at a (1-11) peak for 100 photon max and the Chi metric. (a) shows the modulus cross-sections for all 100 trials. There are 10 trials that were not successful. (b) shows the corresponding Chi metric for all 100 trials. The 10 failed trials are clear from their relatively large modulus errors. (c) shows the corresponding modulus correlation coefficient for all 100 trials. The failed trials are fairly obvious from their low correlation to the true solution.

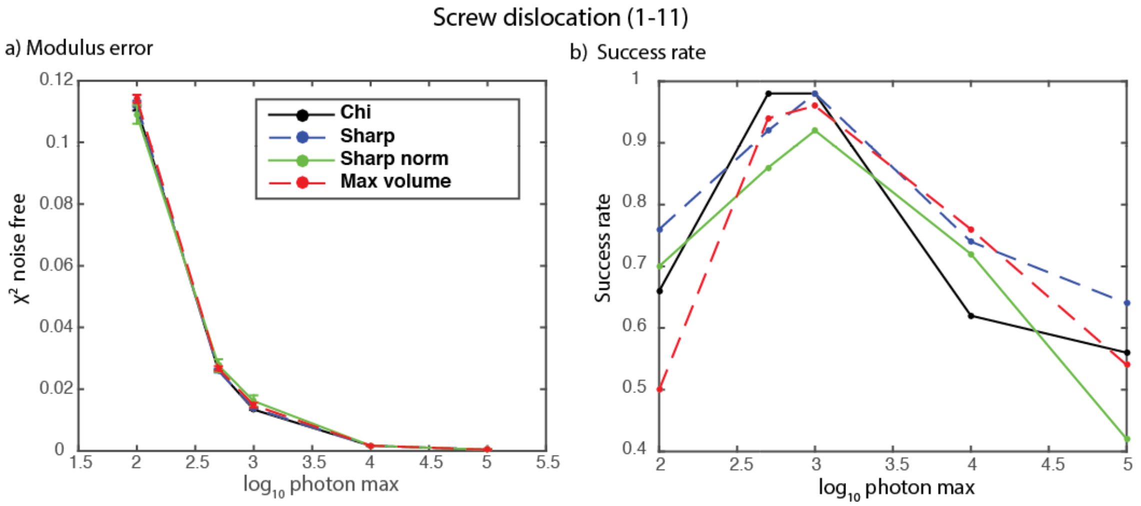

Figure S3. A perfect screw dislocation measured at a (1-11) peak. **(a)** The modulus error (χ^2) is computed with respect to the noise-free data. The error bars correspond to two standard deviations of the modulus error over the 100 trials. Four metrics at five photon maxes are considered. **(b)** A plot of the success rate (over 100 trials) for the four fitness metrics as a function of photon max. The legend is the same as in **(a)**.

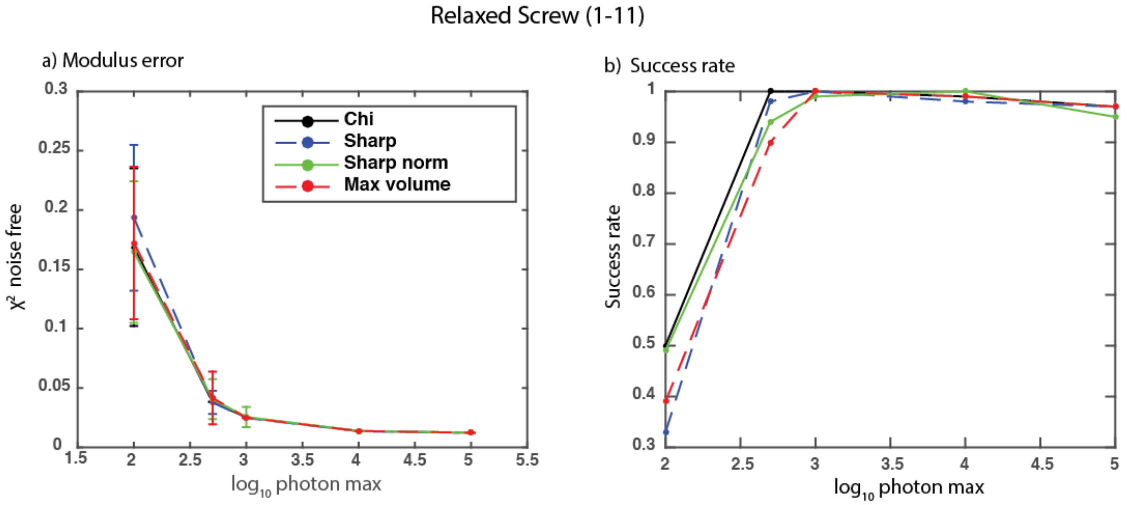

Figure S4. A relaxed screw dislocation measured at a (1-11) peak. **(a)** The modulus error (χ^2) is computed with respect to the noise-free data. The error bars correspond to two standard deviations of the modulus error over the 100 trials. Four metrics at five

photon maxes are considered. **(b)** A plot of the success rate (over 100 trials) for the four fitness metrics as a function of photon max. The legend is the same as in **(a)**.

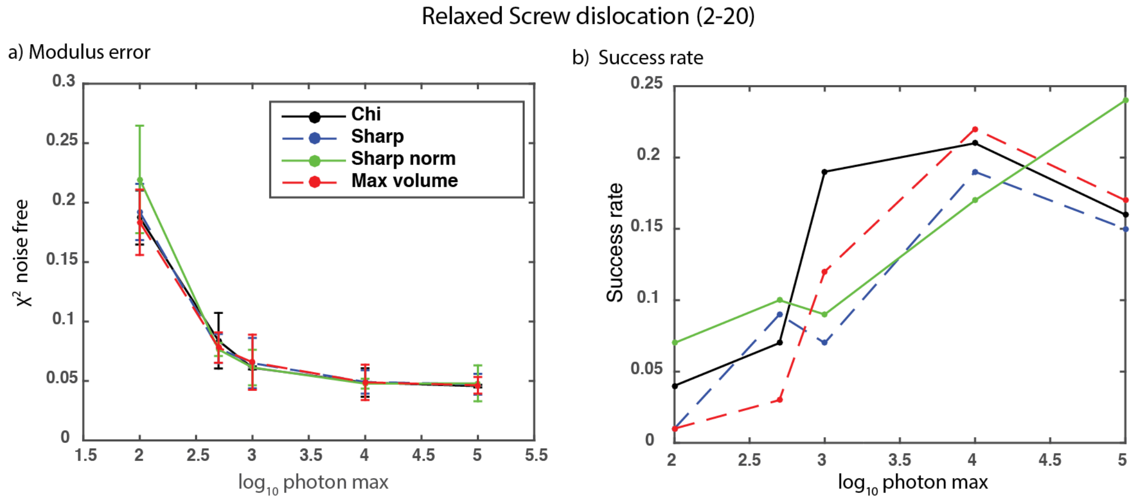

Figure S5. A relaxed screw dislocation measured at a (2-20) peak. **(a)** The modulus error (χ^2) is computed with respect to the noise-free data. The error bars correspond to two standard deviations of the modulus error over the 100 trials. Four metrics at five photon maxes are considered. **(b)** A plot of the success rate (over 100 trials) for the four fitness metrics as a function of photon max. The legend is the same as in **(a)**.

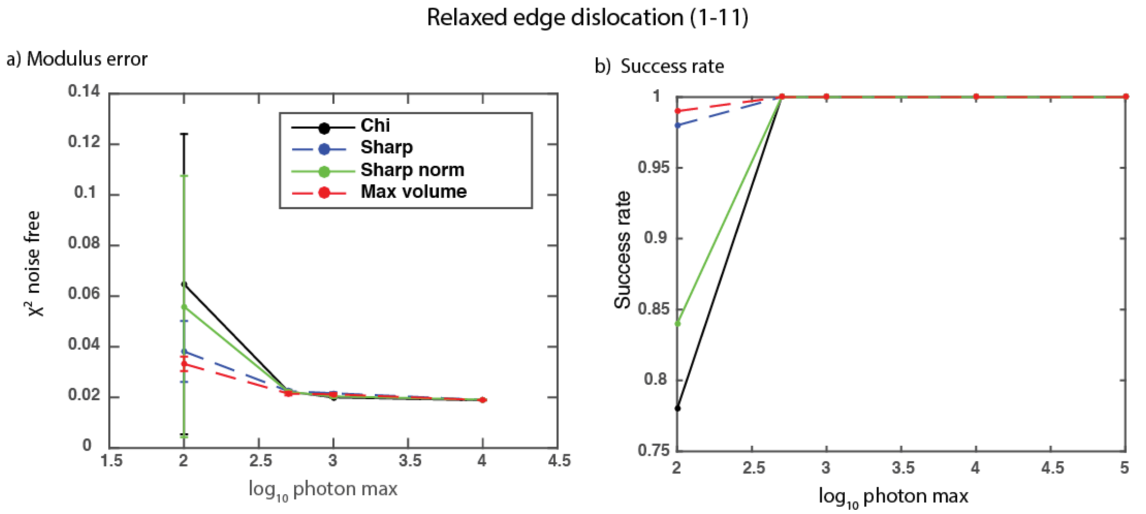

Figure S6. A relaxed edge dislocation measured at a (1-11) peak. **(a)** The modulus error (χ^2) is computed with respect to the noise-free data. The error bars correspond to two standard deviations of the modulus error over the 100 trials. Four metrics at five photon maxes are considered. **(b)** A plot of the success rate (over 100 trials) for the four fitness metrics as a function of photon max. The legend is the same as in **(a)**.

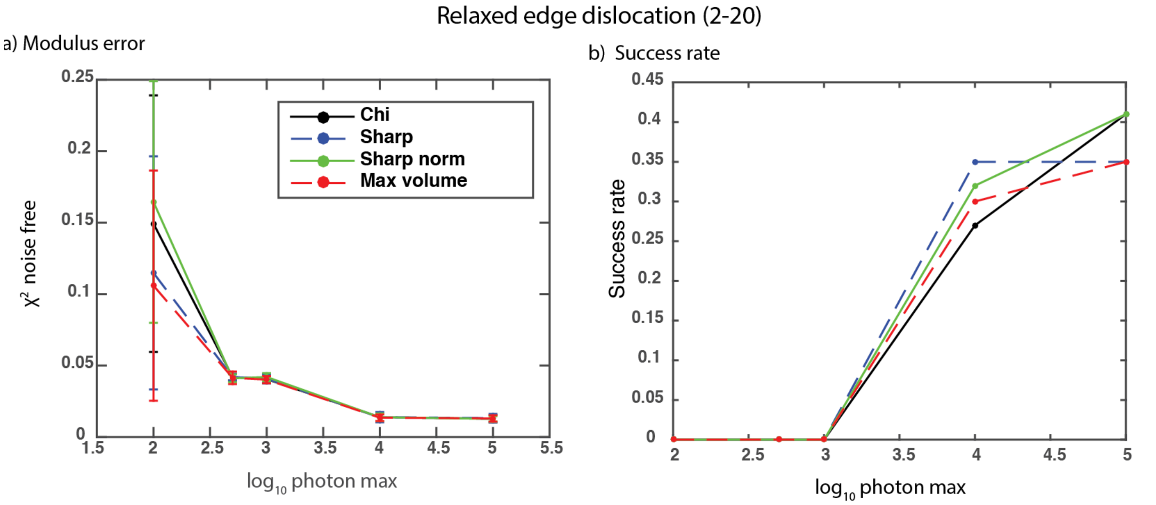

Figure S7. A relaxed edge dislocation measured at a (2-20) peak. **(a)** The modulus error (χ^2) is computed with respect to the noise-free data. The error bars correspond to two standard deviations of the modulus error over the 100 trials. Four metrics at five photon maxes are considered. **(b)** A plot of the success rate (over 100 trials) for the four fitness metrics as a function of photon max. The legend is the same as in **(a)**.

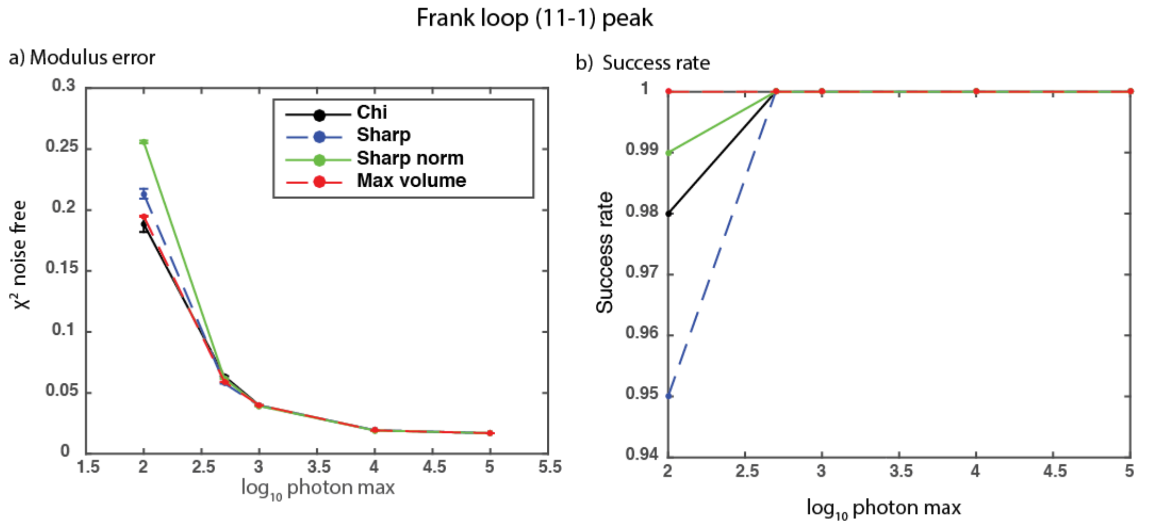

Figure S8. A Frank loop measured at a (11-1) peak. **(a)** The modulus error (χ^2) is computed with respect to the noise-free data. The error bars correspond to two standard deviations of the modulus error over the 100 trials. Four metrics at five photon maxes are considered. **(b)** A plot of the success rate (over 100 trials) for the four fitness metrics as a function of photon max. The legend is the same as in **(a)**.

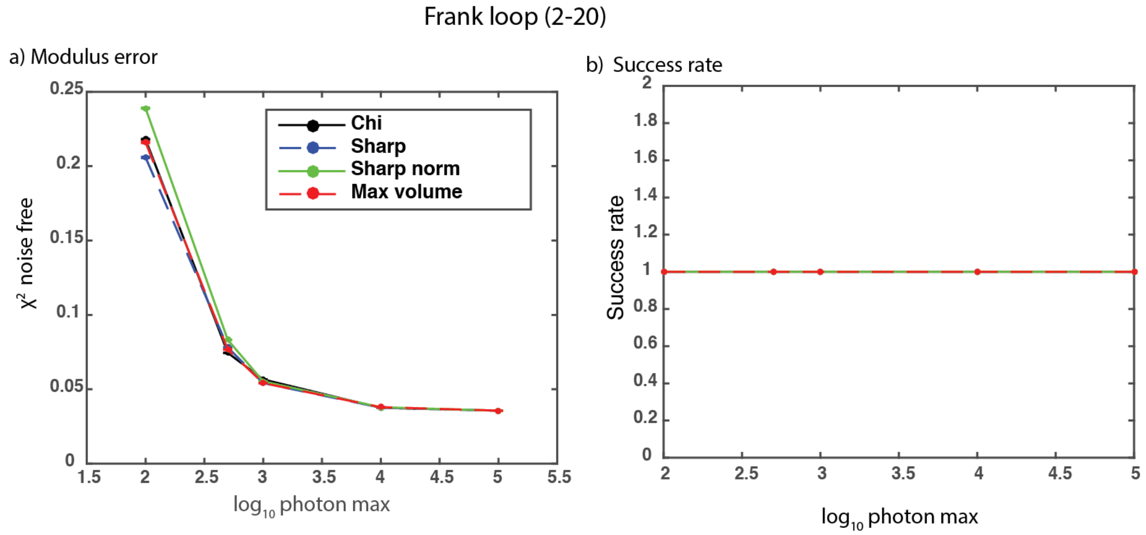

Figure S9. A Frank loop measured at a (2-20) peak. **(a)** The modulus error (χ^2) is computed with respect to the noise-free data. The error bars correspond to two standard deviations of the modulus error over the 100 trials. Four metrics at five photon maxes are considered. **(b)** A plot of the success rate (over 100 trials) for the four fitness metrics as a function of photon max. The legend is the same as in **(a)**.

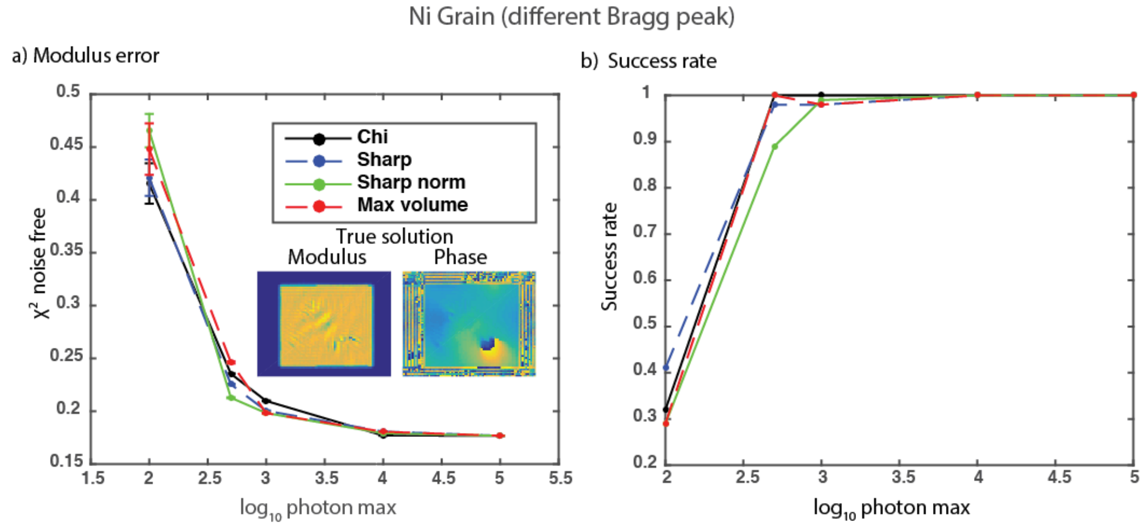

Figure S10. A different Bragg peak from the same defect distribution in a Ni film grain. **(a)** The modulus error (χ^2) is computed with respect to the noise-free data. The error bars correspond to two standard deviations of the modulus error over the 100 trials. Four metrics at five photon maxes are considered. **(b)** A plot of the success rate (over 100 trials) for the four fitness metrics as a function of photon max. The legend is the same as in **(a)**.

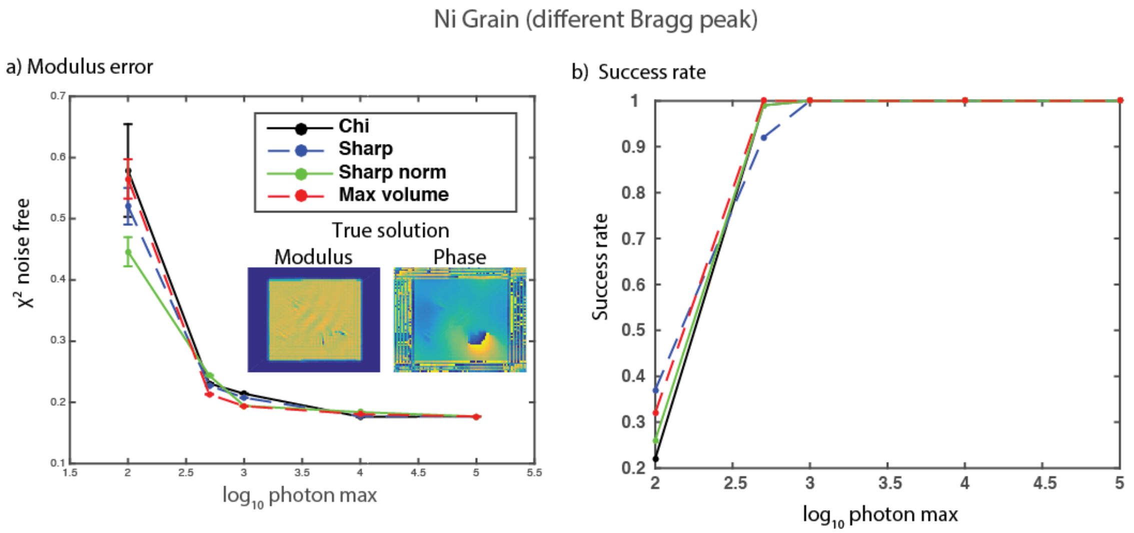

Figure S11. A different Bragg peak from the same defect distribution in a Ni film grain. **(a)** The modulus error (χ^2) is computed with respect to the noise-free data. The error bars correspond to two standard deviations of the modulus error over the 100 trials. Four metrics at five photon maxes are considered. **(b)** A plot of the success rate (over 100 trials) for the four fitness metrics as a function of photon max. The legend is the same as in **(a)**.
